# RNA-Seq Analysis of *Magnaporthe grisea* Transcriptome Reveals the High Potential of ZnO Nanoparticles as a Nanofungicide

**DOI:** 10.3389/fpls.2022.896283

**Published:** 2022-06-10

**Authors:** Reza Ghamari, Asadollah Ahmadikhah, Masoud Tohidfar, Mohammad Reza Bakhtiarizadeh

**Affiliations:** ^1^Department of Biotechnology, Faculty of Life Sciences and Biotechnology, Shahid Beheshti University, Tehran, Iran; ^2^Department of Animal and Poultry Science, College of Aburaihan, University of Tehran, Tehran, Iran

**Keywords:** zinc oxide, antifungal activity, RNA sequencing, nanobiofungicide, rice blast

## Abstract

*Magnaporthe grisea* is one of the most destructive pathogen that encounters a challenge to rice production around the worldwide. The unique properties of ZnO nanoparticles (NPs), have high attractiveness as nanofungicide. In the present study, the response of fungi to ZnO NPs was evaluated using RNA sequencing (RNA-seq). Two different aligners (STAR and Hisat2) were used for aligning the clean reads, and the DEseq2 package was used to identify the differentially expressed genes (DEGs). In total, 1,438 and 761 fungal genes were significantly up- and down-regulated in response to ZnO NPs, respectively. The DEGs were subjected to functional enrichment analysis to identify significantly enriched biological pathways. Functional enrichment analysis revealed that “cell membrane components,” “ion (calcium) transmembrane transporter activity,” “steroid biosynthesis pathway” and “catalytic activity” were the contributed terms to fungal response mechanisms. The genes involved in aflatoxin efflux pumps and ribosome maturation were among the genes showing significant up- and down-regulation after ZnO NPs application. To confirm the obtained RNA-seq results, the expression of six randomly selected genes were evaluated using q-RT-PCR. Overall, the RNA-seq results suggest that ZnO NPs primarily act on the fungal cell membrane, but accumulation of ROS inside the cell induces oxidative stress, the fungal catalytic system is disrupted, resulting into the inhibition of ROS scavenging and eventually, to the death of fungal cells. Our findings provide novel insights into the effect of ZnO NPs as a promising nanofungicide for effective control of rice blast disease.

## Introduction

Rice (*Oryza sativa*) is arguably the most widely consumed food crop in the world, and it is a relatively good source of calories for half of the world’s population (Dean et al., 2012). Due to the increasing population of the world and to ensure food security, increasing rice yield potential has become a major challenge (Skamnioti and Gurr, 2009). On the other hand, plant pathogenic fungi can cause devastating crop damage, and every year heavy compensation is paid to decrease plant disease effects, thereby food security (Strange and Scott, 2005; Fisher et al., 2018). Rice blast is one of the most destructive fungal diseases in all rice-growing regions and has a significant negative impact on global rice production (Wang and Valent, 2009). Blast disease is caused by the hemibiotrophic fungus *Magnaporthe grisea*, whose outbreak and intensity indirectly increase the costs of production (Wilson and Talbot, 2009; Martin-Urdiroz et al., 2016). Rice blast disease by up to 30% can reduce the global harvest of rice, so identifying and using sustainable and safe solutions that do not have the limitations of traditional methods are essential to control this disease (Talbot, 2003; Fisher et al., 2012). Nanomaterials, especially metal oxide nanomaterials, are widely used due to their unique capabilities. One of the most vital properties of metal oxide nanoparticles (NPs) is their antifungal activity; therefore, these nanomaterials have been used in different areas, such as nanomedicine, cosmetics, and agriculture (Azam et al., 2012). Among the various metal oxide nanomaterials, zinc oxide is the most valuable material widely used as an antimicrobial agent. Being an antimicrobial agent at a very low concentration is one of the advantages of distinguishing ZnO nanoparticles from other nanomaterials (Doumbia et al., 2015; Hameed et al., 2016; Mishra et al., 2017). In addition, although there are many chemical and physical methods for nanomaterial synthesis, biomaterials such as plants allow the synthesis of nanoparticles by a healthy, economical and environmentally safe method with uniform shape and size (Ogunyemi et al., 2019). Several conventional methods have been used to overcome plant pathogens, each of which has specific limitations. For instance, the use of common fungicides and the high cost of traditional control of plant diseases have harmful environmental and nutritional effects that can even endanger human health (Waard et al., 1993; Horrigan et al., 2002). Therefore, the use of green synthesized nanoparticles is an effective alternative that is safe, highly adaptable to the environment, and economical to control pathogens (Kumar and Yadav, 2009). These nanoparticles have a higher potential in managing plant pathogens than synthetic fungicides and can be the best alternative to traditional fungicides (Park et al., 2006). Based on recent studies, the production of reactive oxygen species, dissolution of Zn^2+^ ions, and damage to the bacterial cell wall have been suggested as mechanisms for ZnO antibacterial activity (Sirelkhatim et al., 2015; Joe et al., 2017; Medina-Velo et al., 2017). Many studies have been performed on the antimicrobial activity of nanomaterials (Simon et al., 2013). However, only a few studies have investigated the effects of metal oxide nanoparticles on fungal pathogens; additionally, there is no study about ZnO nanoparticles against *M. grisea.*

A robust approach to identify how an organism responds to a particular stress condition is to evaluate how it affects genome expression. High-throughput methods of transcriptome study, such as RNA sequencing, can quantify the expression of all genes in an organism at the level of RNA transcripts in response to stress conditions versus normal physiological conditions (Chen et al., 2012). The degree of similarity and difference can be revealed by comparing the effects of different biotic and abiotic conditions. For example, if exposure to nanoparticles causes a specific stress, these changes can be tracked and identified by differential expression (DE) at the transcript level of a particular set of genes. It is possible to identify different physiological and biochemical processes by altering transcription levels in genes with known or predicted functions. Although there are no studies on the response of *M. grisea* to zinc oxide nanoparticles, profiling of gene expression using RNA-seq makes it possible to understand different layers of its response mechanism to these nanoparticles. In this study, ZnO nanoparticles were synthesized with a green method [using green tea (*Camellia sinensis*) leaf extract], and after obtaining ideal ZnO NPs, these nanoparticles were treated on *M. grisea*. Finally, treated samples with ZnO NPs were compared to control samples at the transcriptome level using RNA-seq sequencing technology to identify key pathways and genes as well as potential molecular mechanisms in response to zinc oxide nanoparticles.

## Materials and Methods

### Ethics Statement, Fungal Growth Condition, ZnO Nanoparticle Synthesis

*Magnaporthe grisea* (WDCM ID: 939) was generously provided by the Iranian Research Institute of Plant Protection. Fungi were grown on potato dextrose broth (PDB) and incubated at 28°C. The synthesis of ZnO nanoparticles was performed by the green synthesis method using leaf extract of green tea (*C. sinensis*; Senthilkumar and Sivakumar, 2014) in compliance with national guidelines and legislation. The ZnO NPs were characterized by Fourier transform infrared spectroscopy (FTIR), X-ray diffraction (XRD) and Field Emission Scanning Electron Microscope (FE-SEM). All characterization approaches confirmed the presence of ZnO nanoparticles with optimal size (20–30 nm), shape and uniformity. The minimum inhibitory concentration (MIC) was determined by the broth dilution method (Shukla et al., 2009). To obtain the MIC, fungi were grown on PDB medium with different concentrations of ZnO NPs (1–5 mM). Based on the test, the MIC of ZnO nanoparticles was estimated to be 5 mM (0.4 mg/ml). Therefore, after treatment of fungi with ZnO NPs using the dilution method in PDB medium (Wiegand et al., 2008), total RNA was isolated in two biological replicates for each condition (treatment and control, in total four samples) for paired-end transcriptome sequencing.

### Total RNA Extraction and Transcriptome Sequencing

Total RNA was isolated from approximately 50 mg of fungal crushed tissue using Qiagen RNeasy Mini Kit by following the manufacturer’s protocol. First, to check 28S/18S rRNA band intensity, the purification and quantification of RNA samples were evaluated by 1% agarose gel electrophoresis and using a Nanodrop (Thermo Scientific Nanodrop one/one microvolume UV–Vis spectrophotometer), respectively. All qualified samples were sent to BGI Company (China). In the next step, the quality control of samples was performed using a Bioanalyzer 2100 and RNA 6000 Nano LabChip Kit (Agilent, CA, United States) by the company (Zhu et al., 2018). A 28S/18S ratio above 2, an OD260/280 ratio greater than 1.8 and an integrity number of RNA (RIN) > 8 were the main priorities to select samples for RNA sequencing. Finally, each captured library was loaded on the BGI-seq500 platform, and high-throughput sequencing was performed for each captured library independently to ensure that each sample met the desired average fold coverage. Raw image files were processed by Illumina base calling Software 1.7 for base calling with default parameters, and the sequences of each individual were generated as 100 bp paired-end reads. The raw RNA-Seq data were launched in the Sequence Read Archive (SRA) database with the BioProject accession number PRJNA664016.

### Preprocessing, Quality Control, and Read Trimming

Quality assessment of short reads was performed using FASTQC tools (v0.11.5; Bioinformatics, 2011). Preprocessing and removal of low-quality reads and adapter contamination was carried out using Trimmomatic (version 0.39; Bolger et al., 2014). Chosen options for trimming were TRAILING:20, MAXINFO:120:0.9, and MINLEN:120 (Bakhtiarizadeh et al., 2019). The quality of the reads was reassessed with FastQC after these steps to confirm quality improvements.

### Reads Mapping to Genome and DEGs Analysis

The basic step in RNA-seq studies to identify differentially expressed genes (DEGs) is mapping the reads against the reference genome. Currently, many alignment tools and normalization/statistical methods have been developed for this purpose (Shang et al., 2014). Based on benchmark studies and comparisons of different methods/tools, there is no single method or tool that exceeds the other methods (Baruzzo et al., 2017). The results of these studies have shown that tool selection at each step has a significant impact on the process of interpreting RNA-seq data (Engström et al., 2013). Additionally, combining different methods can be an effective strategy to obtain reliable results (Costa-Silva et al., 2017). In this study, for deprivation of false positive results, the results of two alignment tools (Hisat2 and STAR) were combined. These tools were selected based on their high usability, and the combination and use of overlapping results of these tools can improve the efficiency of DEG identification (Engström et al., 2013; Baruzzo et al., 2017; Costa-Silva et al., 2017; Everaert et al., 2017). Statistical DEseq2 R package was used to extract the raw read counts (uniquely mapped reads) related to each gene using the reference genome (GCA_000002495.2; Love et al., 2014). Therefore, two separate combinations were considered to identify DEGs, including Hisat + DEseq2 and STAR + DEseq2. Finally, only the set of genes identified by the two methods as DEGs were considered for downstream analysis (based on fold change >|2| and adjusted value of *p* < 0.05).

### Gene Regulatory Network Analysis

Understanding cellular function requires understanding the interactions among proteins (Sinz, 2018). STRING (the Search Tool for the Retrieval of Interacting Genes/Proteins) is a repository displaying information about physical and functional associations of over 9 million proteins from more than 2,000 organisms (Szklarczyk et al., 2016). The putative networks of up-regulated and down-regulated DEGs were constructed using STRING separately, and the nodes without interactions were hidden. Cytoscape (version 3.8.2) was used to visualize and analyze the different aspects of the networks (Nepusz et al., 2012). To do this, ClusterONE (Clustering with Overlapping Neighborhood Expansion, version 1.0) and the CytoHubba plugin in Cytoscape were applied to identify subnetworks/modules and hub genes in the constructed PPI network, respectively. The ClusterONE plugin discovers density and possibly overlapping modules in the networks. CytoHubba allows the use of several topological analysis algorithms to detect the hub genes in the network. In this study, closeness, MCC (maximal clique centrality), and MNC (maximum neighborhood component) algorithms were used to identify hub genes in the constructed PPI network.

### Gene Ontology and KEGG Pathway Enrichment Analysis

To obtain further insight into different metabolic processes, functional enrichment analysis, including gene ontology (GO) and Kyoto Encyclopedia Genes and Genomes (KEGG) pathway analyses, was performed for the up- and down-regulated genes. For this aim, g.profiler was used as a web-based tool (using adjusted value of *p* ≤ 0.05; Raudvere et al., 2019). This analysis provides a system for hierarchically classifying information about the significant biological functions and pathways enriched in up- or down-regulated genes.

### q-RT-PCR Validation of Selected DEGs

For RNA-seq results validation, six genes were randomly selected from the significant DEGs list. RNA was extracted from the fungi treated with zinc oxide nanoparticles under the same conditions. cDNA was synthesized using a Biotechrabbit cDNA Synthesis kit according to the company protocol. The primers were designed using SnapGene software (v.5.2.3). Primer information is presented in Supplementary Table 1. qPCR was performed using a Corbett Rotor-Gene 6000 HRM Real Time PCR Machine with Cyber Green Master Mix (RealQ Plus Master Mix Green, Amplicon) with three biological replications for each gene. The actin gene was used as a housekeeping gene (Ray et al., 2016). To compare the relative expression of genes, the ΔΔC_T_ method was used. Finally, the mean 2^−ΔΔCT^ value of genes was changed to fold change (FC) to allow comparison between RNA-seq and q-RT-PCR results.

## Results

### Antifungal Effect of ZnO NPs on *Magnaporthe grisea*

**Figure 1:**
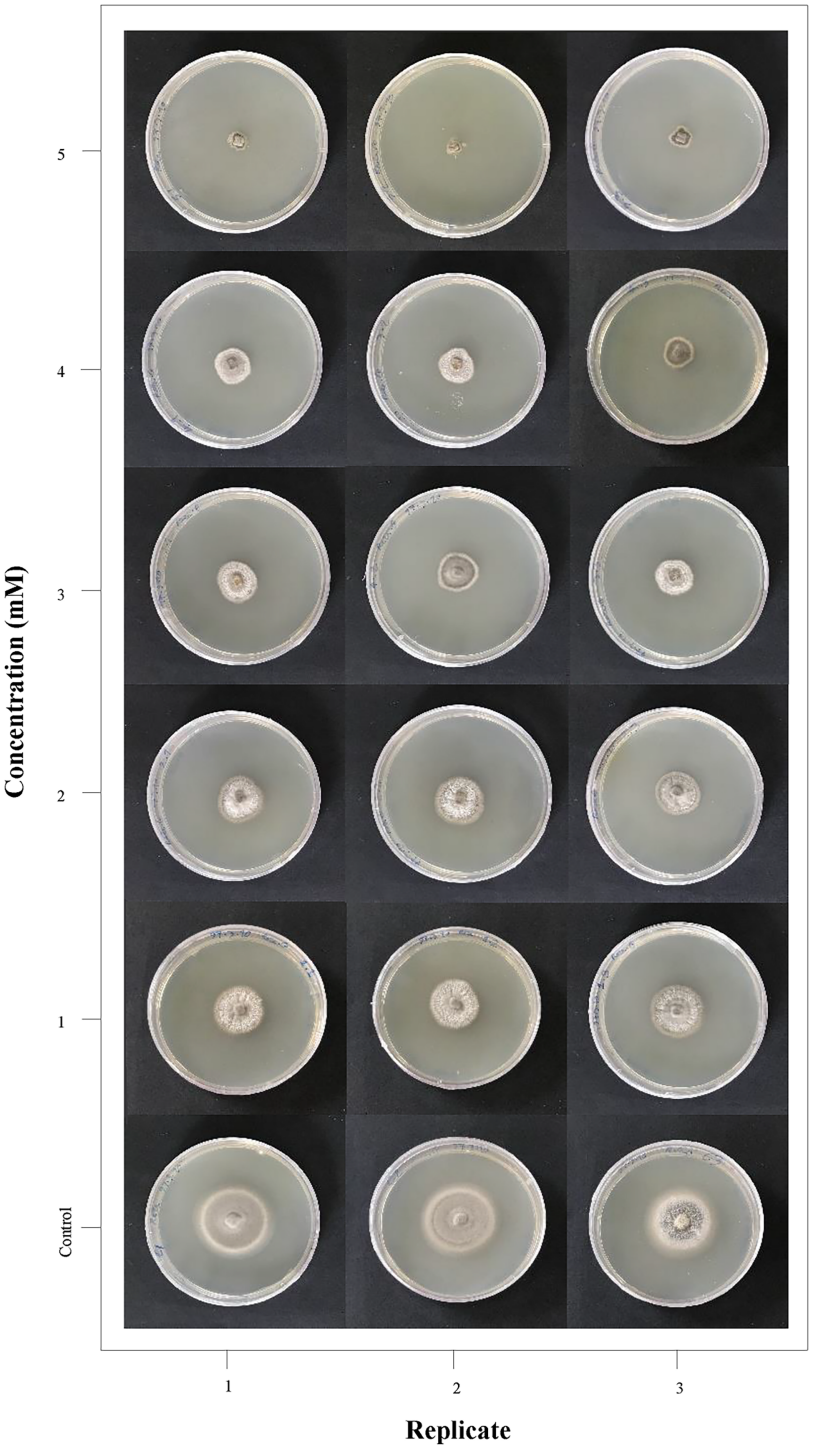
Antifungal activity of ZnO nanoparticles (NPs) synthesized using the *Camellia sinensis* leaf extract. *Magnaporthe grisea* growth on different concentrations of medium containing ZnO NPs (0–5 mM) is represented in three replicates for visual comparison.

The antifungal efficacy of ZnO NPs was tested at different concentrations (1–5 mM) against *M. grisea*. Fungal growth on potato dextrose agar (PDA) medium and antibiogram test results showed that ZnO NPs had significant growth inhibition. The variance analysis and means comparison of fungal growth over 10 days showed that significant growth inhibition was obtained at a 5 mM (0.4 mg/ml) concentration of ZnO NPs on the 10th day (Figure 1). The results of variance analysis and Duncan’s multiple range test are presented in Supplementary Table 2. All results showed that the fungus did not grow in PDB medium in the presence of 5 mM ZnO NPs, but after the transfer of the fungal inoculum from the liquid medium (PDB) to the solid medium (PDA), it had slight growth; at this concentration, the lowest growth rate was observed in comparison to other concentrations (1–4 mM); therefore, the 5 mM concentration was selected as the MIC and used for the treatment of fungi in the transcriptome study.

### RNA-Seq Raw Data Generation

In total, 94,030,238 paired-end raw reads were generated for four samples (two replicates of control and treated samples). After trimming, only 504 reads were filtered, and 94,029,734 reads were maintained in the initial short read batch, indicating high efficiency of sequencing and data quality. As described, two aligners were used for clean read mapping against the reference genome; on average, 89% of the clean reads were mapped to the genome, and 85.6% of these reads were mapped uniquely. A summary of the obtained RNA sequencing data sets and mapping rate of clean reads are shown in Table 1.

**Table 1:** Different statistics of sequence quality and mapping rate for four samples of the *M. grisea* transcriptome.

Sample	Total raw reads	Cleaned reads	Hisat2 overall mapped (%)	STAR overall mapped (%)	Hisat2 unique mapped (%)	STAR unique mapped (%)
Control-1	22,356,230	22,356,101	20,175,351 (90)	20,082,482 (88.4)	18,896,537 (84)	19,430,400 (86)
Control-2	21,890,182	21,890,104	19,796,774 (90)	19,700,115 (88.5)	18,560,881 (84)	19,055,551 (87)
Treatment-1	22,822,245	22,822,093	20,718,338 (90)	20,683,712 (89.2)	19,561,933 (84)	20,142,222 (88)
Treatment-2	26,961,581	26,961,436	24,488,120 (90)	24,449,990 (89.1)	23,087,701 (84)	23,797,283 (88)

### Differential Gene Expression Analysis

**Figure 2:**
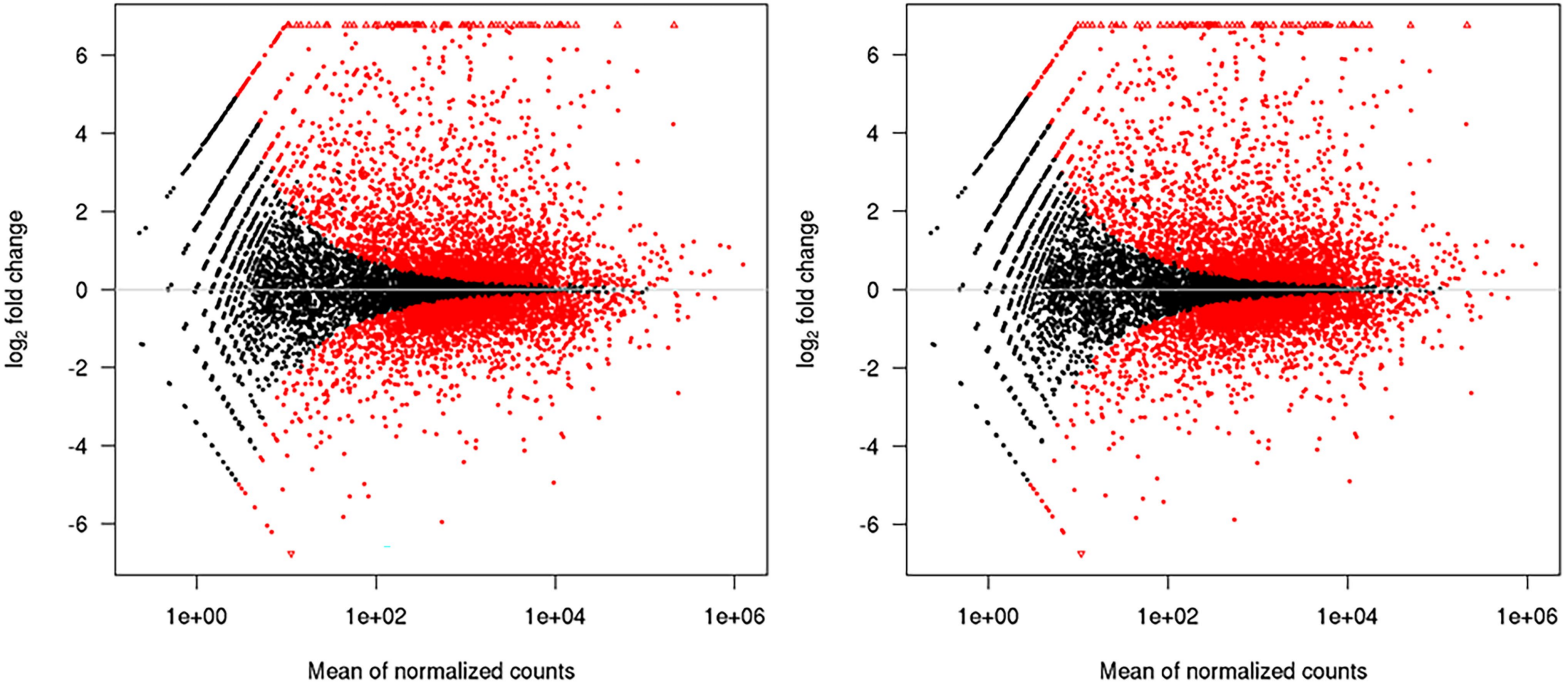
MA plots generated by the DEseq2. The left and right plots are related to the Hisat2 + DEseq2 and STAR + DEseq2 data sets, respectively. The high similarity of the plots indicates that the obtained data from the two methods have the same expression pattern.

**Figure 3:**
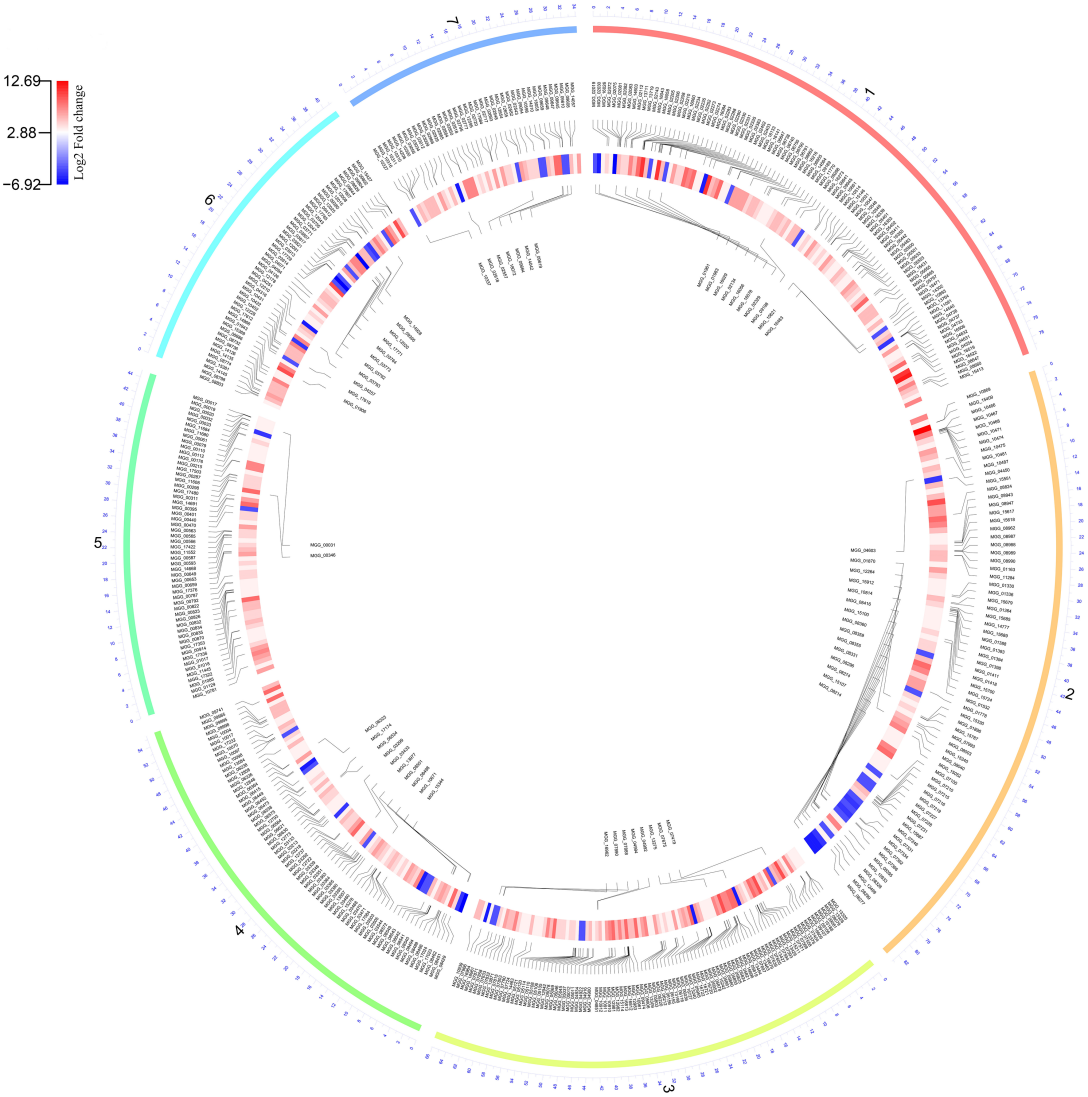
The Circos plot shows the pattern of gene expression in fungi during the zinc oxide nanoparticle response. The outer layer shows seven fungal chromosome numbers. The inner layer and central layer show the upregulated and downregulated genes, respectively. The expression pattern is illustrated by a circular heatmap of differentially expressed genes (DEGs).

Comparative transcriptome analysis of *M. grisea* fungi in response to ZnO NPs was performed by two approaches: Hisat2 + DEseq2 and STAR + DEseq2 (Bakhtiarizadeh et al., 2019). In the first approach, 6,194 and in the second approach, 6,210 genes were identified as DEGs, and by combining the results of two approaches, 1,438 and 761 genes were identified as up- and down-regulated genes, respectively. The comparison of MA plots in the two methods (Hisat2 + DEseq2 and STAR + DEseq2) indicates a slight difference in the expression pattern (Figure 2). The list of identified DEGs is provided in Supplementary Table 3. To visualize the expression pattern and distribution of DEGs across chromosomes, a circos plot was used as a comprehensive plot (Figure 3).

### Functional Enrichment Analysis

**Figure 4:**
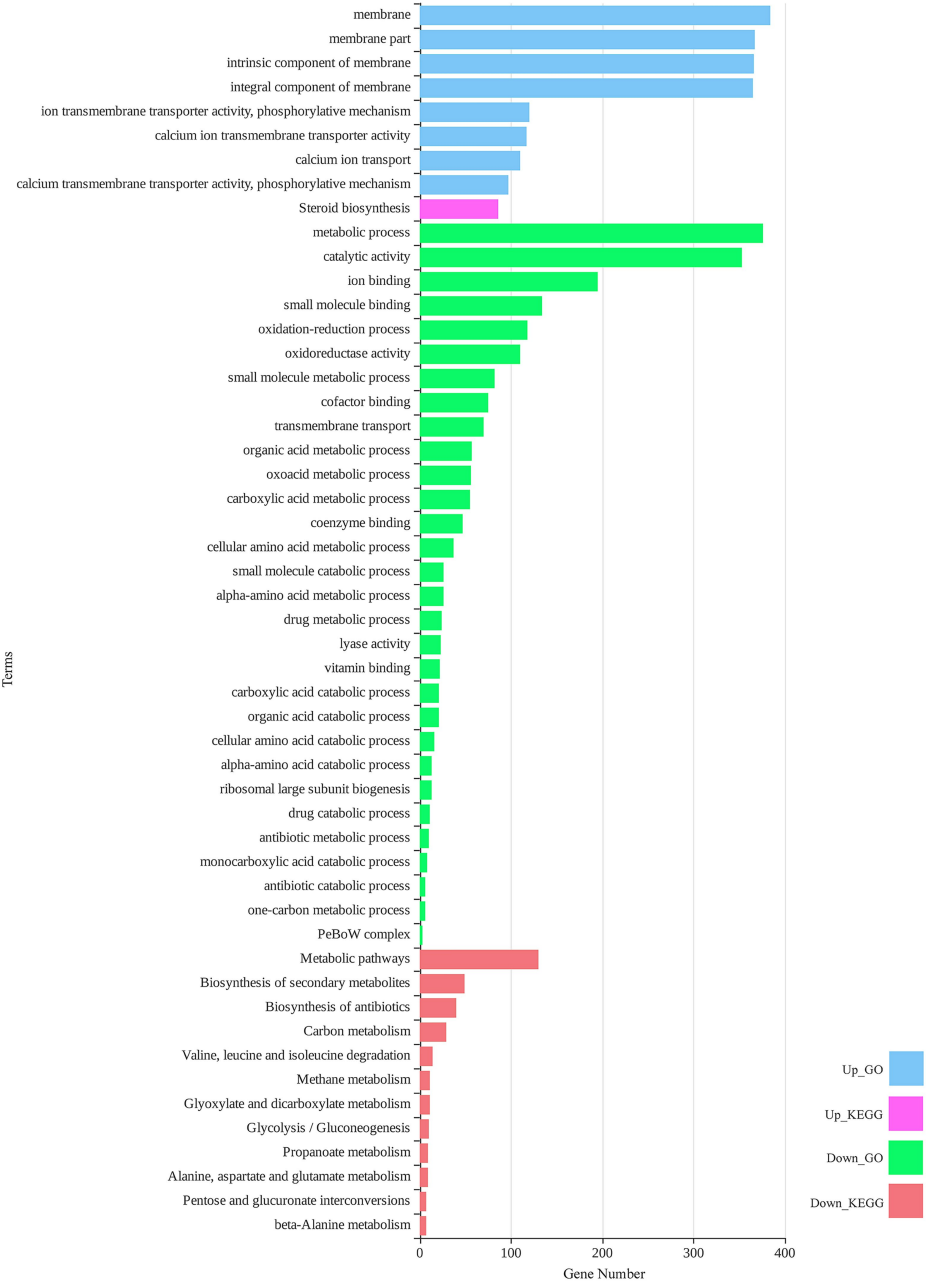
Total significant (value of *p* < 0.05) gene ontology (GO) terms and Kyoto Encyclopedia Genes and Genomes (KEGG) pathways for differentially expressed genes of *M. grisea* in the presence of ZnO NPs.

DEGs were considered a source to understand different layers of biological behavior in response to zinc oxide nanoparticles. To determine the metabolic processes and response pathways of *M. grisea* to ZnO nanoparticles, functional enrichment analysis was performed (Figure 4). The GO enrichment of up-regulated DEGs revealed eight significant GO terms. In the cell component category, the total enriched terms were relevant to fungal cell “membrane parts,” such as integral and intrinsic components of the membrane. “Calcium transmembrane transporter activity, phosphorylative mechanism,” “calcium ion transmembrane transporter activity,” and “ion transmembrane transporter activity, phosphorylative mechanism” were the three enriched terms in the molecular function category. “Calcium ion transport” was the only term in the biological process category. The KEGG pathway analysis of up-regulated DEGs showed only one significant pathway, “steroid biosynthesis.” In the case of down-regulated DEGs subjected to GO enrichment, “metabolic process” and “oxidation–reduction process” were significant terms in the biological process category. “Catalytic activity,” “ion binding,” “small molecule binding,” and “oxidoreductase activity” were the most important enriched terms in the molecular function category. In KEGG pathway analysis of down-regulated DEGs, 12 significant pathways were identified, which had close associations with GO terms. “Metabolic pathways,” “biosynthesis of secondary metabolites,” and “biosynthesis of antibiotics” were the three top pathways. Detailed results of functional enrichment analysis are provided in Supplementary Table 4.

### Network Analysis and Hub Genes Identification

**Figure 5:**
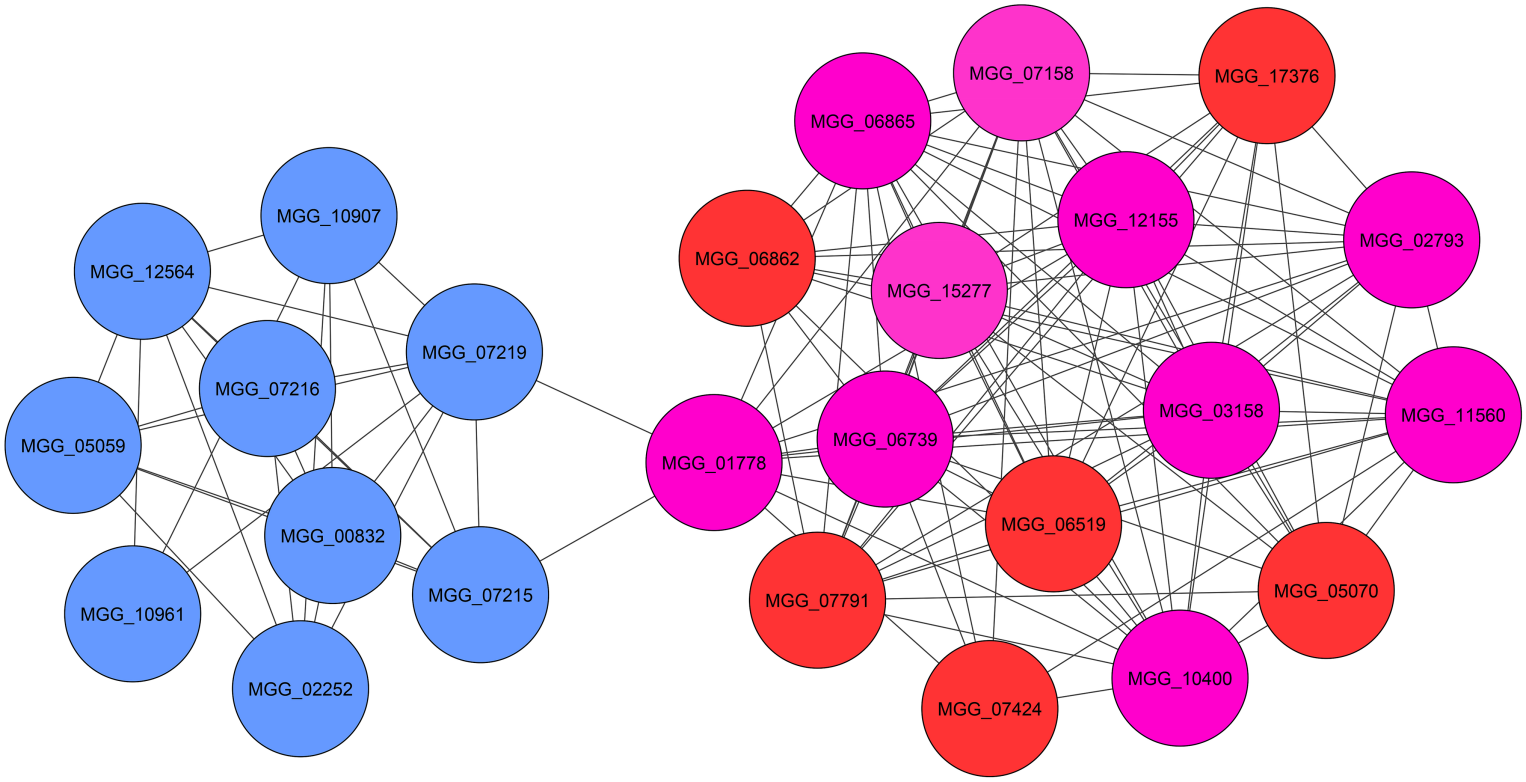
The significant modules (blue and red) and identified hub genes (purple) associated with up-regulated DEGs of *M. grisea* fungi in response to ZnO NPs.

**Figure 6:**
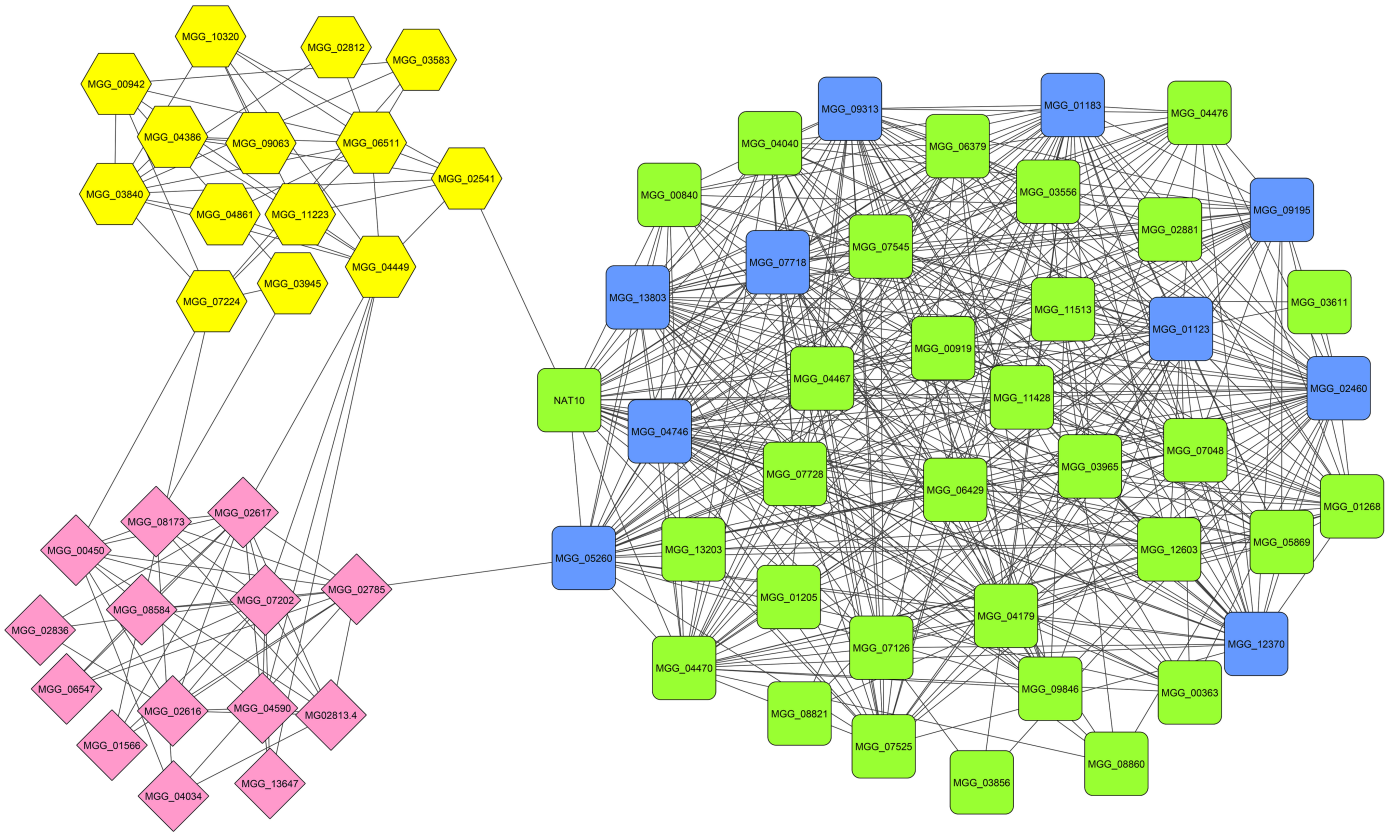
The significant modules (green, yellow, and pink) and identified hub genes (blue) associated with down-regulated DEGs of *M. grisea* fungi in response to ZnO NPs.

To comprehend the functional interaction and identify hub genes involved in the fungal response to zinc oxide NPs among the up- and down-regulated genes, a PPI network was established using the STRING tool. STRING is a database of known and predicted protein–protein interactions for a large number of organisms, including functional and physical interactions. Initial results from STRING showed that the network of up-regulated DEGs had 1,434 nodes and 410 edges. Additionally, the network of down-regulated DEGs contained 760 nodes and 902 edges. Integrating information into networks can lead to the conception of a functional network and functional modules and can also provide a broad understanding of the function of genes, according to studies of protein clustering within functional modules (Mozduri et al., 2018). Therefore, the functional modules as described above were obtained using the clusterONE plugin. In the up-regulated gene network, two significant modules were identified: the first module consisted of 16 genes, and the second module contained nine genes (Figure 5). Additionally, three significant modules were identified in the down-regulated gene network with 41, 14, and 14 genes (Figure 6). Using the cytoHubba plugin in the Cytoscape tool for each set of DEGs, 10 genes were identified as hub genes in the whole networks. Three different algorithms (closeness, MCC, MNC) were used to identify hub genes that eventually overlapped genes in these three algorithms and were reported as hub genes. To determine the function of the obtained hub genes, the BioMart database was used. The functions of the identified hub genes in the up-regulated genes included “Aflatoxin efflux pumps,” “Glycosyl hydrolase family 76 protein,” “Pumilio-family RNA binding repeat protein,” “GPI-anchored cell wall beta-1,3-endoglucanase EglC,” and “Taurine catabolism dioxygenase TauD.” In the set of down-regulated genes, the functions of most of the hub genes were related to translation, such as “PrerRNA processing protein Rrp12,” “Pescadillo homolog,” “Ribosome biogenesis protein YTM1,” “DOM34-interacting protein 2,” “ATP-dependent RNA helicase DRS1,” “rRNA 2’-O-methyltransferase fibrillarin,” “Ribosome biogenesis protein ERB1,” and “MKI67 FHA domain-interacting nucleolar phosphoprotein.”

### Verification of DEGs Through q-RT-PCR

**Figure 7:**
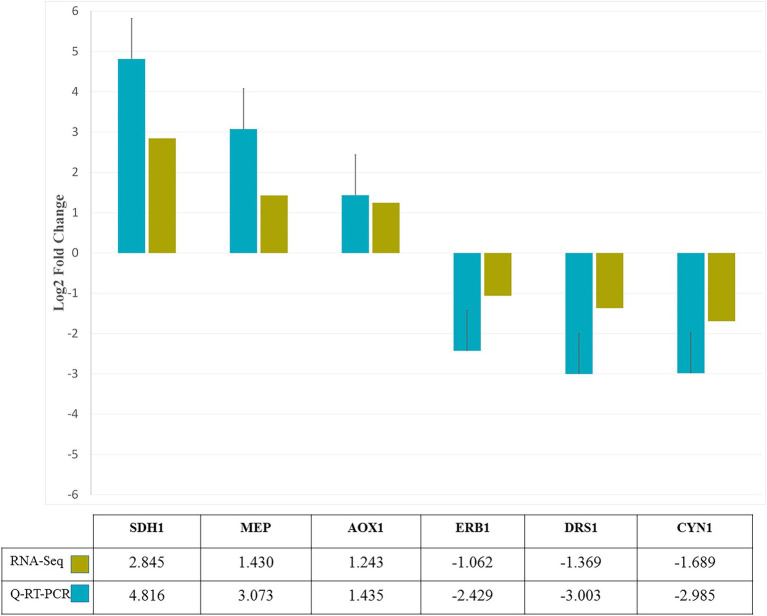
q-RT-PCR confirmation of six genes randomly selected from RNA-Seq analysis. Based on log2-fold change levels, the expression patterns of six genes in response to zinc oxide nanoparticles were similar.

To confirm the expression pattern of DEGs obtained from RNA-seq analysis, six genes were selected randomly and evaluated by q-RT-PCR. Comparison of log2FC values of these genes in RNA-seq and qRT-PCR revealed similarity of gene expression profiles (Figure 7). The similarity of the expression pattern between these two independent experiments indicates that the RNA-seq results are reliable. All q-RT-PCR verification results are provided in Supplementary Table 5.

## Discussion

In this study, RNA-seq-based transcriptome profiling of *M. grisea* was performed under zinc oxide nanoparticle stress as nano-biofungicides. Also, the RNA-seq results were validated by comparing the expression patterns of selected genes using q-RT-PCR, all 6 selected genes had similar expression pattern in two approaches. Our first goal was to recognize possible molecular responses during the growth-inhibitory impacts of zinc oxide NPs to develop mutual strategies in fungi and plants to overcome rice blast outbreaks. To escape false positives, the results of two pipelines with two different aligners (STAR and Hisat2) were merged. Intersection of these methods led to the identification of 1,438 and 761 DEGs. The results of functional enrichment analysis revealed that most of the differentially expressed up-regulated genes were related to membrane, intrinsic and integral components of the membrane, ion (calcium) transmembrane transporter activity, phosphorylative mechanism, calcium ion transport, and steroid biosynthesis. These results indicated that most of the significantly enriched GO terms and KEGG pathways were directly or indirectly related to membrane. Pathogens to achieve a successful infection process must be able to overcome antifungal treatment. Using efflux pumps is a basic way that fungi use to counteract the accumulation of intercellular toxins. In response to chemiosmotic gradients, ATP-binding transporters and the major facilitator superfamily (MFS) remove many toxic compounds across the membrane (Coleman and Mylonakis, 2009). Adaptation of pathogens to abiotic stress requires cellular ion homeostasis, but the presence of zinc oxide nanoparticles interrupts intercellular ion balance (Brini and Masmoudi, 2012). Ion-mediated signaling and their contribution to defense responses are clear. For example, the influx of Ca^2+^ is involved in ROS production, anion efflux, and Ca^2+^-dependent protein kinase cascades, which affect gene expression level (Boudsocq et al., 2010; Jeworutzki et al., 2010). As explained, in the up-regulated DEGs, there was a significant KEGG pathway, biosynthesis of steroids, an extensive group of natural macromolecules that play an essential role in the cell membranes of animals, plants and fungi. The most widely recognized steroid component in fungi is ergosterol (Dupont et al., 2012). Mitochondria is the site of steroidogenesis which is initiated at the inner mitochondrial membrane (IMM), where the conversion of cholesterol to pregnenolone occures. The pregnenolone later enters ER where further enzymatic reactions occur. In other hand, fungi serve as important sources of steroids, indicating the importance of steroids for fungal cell homeostasis or structural purposes. The ZnO nanoparticles are harmfull for fungal whole cell and damage to membranes such as mitochondrial membranes. Therefore, it seems that the genes involved in the steroids biosynthesis which are up-regulated in presense of ZnO nanoparticles, try to enhance the steroids synthesis to overcome the decreased content of them due to the damage of inner mitochondrial membrane. As an example of steroids, ergosterol is a vital compound in the cell membrane structure of fungi that has a similar function to cholesterol in animal cells, and since it affects the fluidity and permeability of the membrane, it assumes significant functions in fungal growth reproduction and stress response (Kodedová and Sychrová, 2015). In the up-regulated genes ontology results, according to the intersection size (Supplementary Table 4), it can be seen that the function of most genes is related to the membrane. Along with these results, by predicting the steroid biosynthesis pathway as the only significant pathway in the presence of zinc oxide nanoparticles, it can be suggested that due to electrostatic interaction, at first the membrane is strongly affected by the nanoparticles. Indeed, ergosterol is the most abundant sterol in fungal cell membranes, where it regulates permeability and fluidity. Because of its crucial functions, unique structural properties, and particular biosynthetic steps, ergosterol is the target of the majority of available antifungals (Da Silva Ferreira et al., 2005). Also, fungi transcriptionally upregulates the expression of ergosterol biosynthesis pathway genes when exposed to antifungal agents that target ergosterol biosynthesis that finally upstream inhibition overrode the effects of downstream inhibition on the ergosterol biosynthesis pathway (Hu et al., 2018). For example, the Azole class of antifungals act by inhibiting the C14α demethylation of lanosterol in fungi, which interferes with the synthesis of the sterol components of the fungal membrane.

Additionally, in the down-regulated gene set, metabolic process, catalectic activity, ion binding, oxidation–reduction process, small molecule binding, and oxidorudactase activity were identified as significant GO terms. Metabolic pathways, biosynthesis of secondary metabolites and biosynthesis of antibiotics were the three most significant KEGG pathways in the down-regulated DEGs. These results indicated that the response mechanisms of *M. grisea* to zinc oxide NPs can be regulated by complex pathways and genes.

From the GO enrichment and KEGG results, it can be inferred that most of the terms appeared under zinc oxide NPs stress condition, corroborate various studies under different stress conditions (Phule et al., 2019). Various mechanisms have been proposed for the antifungal action of nanoparticles, including induction of reactive oxygen species (ROS) production, cell wall damage and lipid peroxidation of the cell membrane mediated by NPs attachment due to their large surface area and electrostatic force, and finally the release of antifungal/antimicrobial ions (Malandrakis et al., 2019, 2020). Any disturbance in the normal redox state of cells can lead to the production of free radicals and oxidative stress. Accumulating these free radicals inside the cell can damage macromolecules, including lipids, proteins, and nucleic acids (Uttara et al., 2009; Pizzino et al., 2017; Goswami, 2020). The production of ROS and the emergence of oxidative stress by activating the ATM-chk2 signaling pathway leads to double-stranded DNA breaks that eventually cause mutagenesis and cell death (Maréchal and Zou, 2013; Howard et al., 2020). In general, nanoparticles with antifungal/antimicrobial properties in the vicinity of the cell affect mitochondrial respiration, mitochondrial apoptosis, activation of the NADPH oxidative system, alteration of calcium homeostasis, and depletion of antioxidant enzymes, all of which are associated with tissue damage (Lobo et al., 2010; Manke et al., 2013). The essential role of calcium in intracellular and extracellular signaling cascades and its extensive dynamic activity indicate the key role of calcium in cell death and the life cycle. There is moreover evidence that calcium signaling pathways affect signaling pathways of ROS production. The mitochondrial metabolic status determines the level of ROS by affecting calcium signaling pathways (Adam-Vizi and Starkov, 2010). Since calcium promotes ATP synthesis by subduing enzymes of the Krebs cycle and oxidative phosphorylation in mitochondria, calcium homeostasis is impaired in the presence of NPs, and when mitochondria are overloaded with calcium, ROS production can be increased independently of the metabolic state (Zorov et al., 2014; Görlach et al., 2015). Although reactive oxygen species were initially considered to be adverse byproducts of aerobic metabolism, evidence suggests that low levels of ROS are involved in various cellular processes, including cell growth and death. There are various studies that show the mutual interaction of ROS and the calcium signaling network, which seems to have a key role in regulating cellular signaling pathways. However, stress can affect the performance of each of these systems and lead to fungal cell death (Csordás and Hajnóczky, 2009). The accumulation of ROS and oxidative stress due to imbalances in production and the ability of biological systems to detoxify ROS can have potentially destructive biological responses. To respond to ROS overloading, cells can enable enzymatic and nonenzymatic antioxidant systems (Pizzino et al., 2017). Superoxide dismutase (SOD), catalase and glutathione peroxidase are natural enzymes that provide intracellular self-defense to balance ROS levels (Tian et al., 2017; Cui et al., 2019). A hierarchical model was presented to illustrate the mechanism of oxidative stress caused by nanoparticles (Manke et al., 2013). According to this model, cells respond to oxidative stress through antioxidant enzyme systems in the presence of nanoparticles. Under slight oxidative stress, the transcription of phase II antioxidant enzymes is mediated by nuclear factor (erythroid-derived 2)-like 2 (Nrf 2) induction. However, toxic levels of oxidative stress cause mitochondrial membrane damage and disruption of the electron transport chain, ultimately leading to cell death by inducing ROS accumulation and depletion of antioxidants in the presence of engineered nanoparticles. Obviously, the results of all analyses confirmed the presented facts. However, identifying the mechanism of NPs action in more detail decodes the different layers of fungi in response to zinc oxide nanoparticles.

The functions of annotated hub genes in the up-regulated DEGs were “Aflatoxin efflux pump (MGG_01778),” “Glycosyl hydrolase family protein (MGG_02793),” “Pumilio-family RNA binding repeat protein (MGG_03158),” “GPI-anchored cell wall beta-1,3-endoglucanase (MGG_10400)” and “Taurine catabolism dioxygenase (MGG_11560).” These genes are directly and indirectly involved in membrane structure and translation levels of cell. Aflatoxin efflux pumps allow microorganisms to regulate their internal environment by removing toxic substances, including antimicrobial agents. Glycosyl hydrolase family proteins are a widespread group of enzymes that hydrolyse the glycosidic bond between two or more carbohydrates. Pumilio-family RNA binding repeat protein is a nucleolar protein required for prerRNA processing involved in the production of 18S rRNA and assembly of the small ribosomal subunit. GPI-anchored cell wall beta-1,3-endoglucanase is an enzyme with glucanosyltransferase activity that is involved in fungal cell wall organization. Taurine catabolism dioxygenase refers to a protein domain that in many fungi is used to breakdown taurine (2-aminoethanesulfonic acid) as a source of sulfur under stress conditions.

From the 10 top identified hub genes in down-regulated DEGs, eight of them were annotated, including “pre-rRNA processing protein (MGG_01123),” “pescadillo homolog (MGG_01183),” “ribosome biogenesis protein YTM1 (MGG_02460),” “DOM34-interacting protein (MGG_04746),” “ATP-dependent RNA helicase DRS1 (MGG_07718),” “rRNA 2’-O-methyltransferase fibrillarin (MGG_09195),” “ribosome biogenesis protein ERB1 (MGG_09313),” and “MKI67 FHA domain-interacting nucleolar phosphoprotein (MGG_12370).” Pre-rRNA processing protein is associated with GSP1, which is required for nuclear export of both pre-40S and pre-60S ribosomal subunits. It is required for the late maturation of the 18S and 5.8S rRNA of the pre-40S ribosomes and for maturation of the 25S and 5.8S rRNA of the pre-60S ribosomes (Oeffinger et al., 2004). Pescadillo homolog is a component of the PeBoW complex, which is required for maturation of 28S and 5.8S ribosomal RNAs and formation of the 60S ribosome (Lapik et al., 2004; Lerch-Gaggl et al., 2007). The ribosome biogenesis protein YTM1 is a part of the NOP7 complex, which is required for maturation of 25S and 5.8S ribosomal RNAs and formation of the 60S ribosome (Miles et al., 2005; Tang et al., 2008; Baßler et al., 2010). DOM34-interacting protein involved in protein translation, together with HBS1, may function in recognizing stalled ribosomes and triggering endonucleolytic cleavage of the mRNA, a mechanism to release nonfunctional ribosomes and degrade damaged mRNAs. The complex formed by DOM34 and HBS1 has ribonuclease activity toward double-stranded RNA substrates but does not cleave single-stranded RNA. It acts as an endonuclease, has no exonuclease activity and increases the affinity of HBS1 for GTP but not for GDP. Additionally, it promotes G1 progression and differentiation and is involved in mitotic and meiotic cell divisions (Doma and Parker, 2006; Lee et al., 2007; Graille et al., 2008). ATP-dependent RNA helicase DRS1 is involved in ribosome assembly. rRNA 2’-O-methyltransferase fibrillarin; S-adenosyl-L-methionine-dependent methyltransferase that has the ability to methylate both RNAs and proteins. The ribosome biogenesis protein ERB1 is a component of the NOP7 complex, which is required for maturation of 25S and 5.8S ribosomal RNAs and formation of the 60S ribosome (Pestov et al., 2001). MKI67 FHA domain-interacting nucleolar phosphoprotein function is related to RNA binding. The results of the functional analysis of hub genes and GO enrichment analysis provide a plan for how nanoparticles influence the cell. After binding nanoparticles to the cell surface due to electrostatic properties, the membrane is affected, and the cell upregulates the expression of genes involved in membrane organization. By maintaining the presence of nanoparticles, disrupting the electron transport chain and downregulating the expression of genes involved in catalytic activity, ROS accumulate inside the cell, and oxidative stress occurs, which damage macromolecules, eventually leading to fungal cell death.

## Conclusion

The findings of this study provide a comprehensive view of the transcriptional responses to Zinc Oxide nanoparticles in *M. grisea* fungi. Among the DEGs with up-regulated expression in response to nanoparticles were genes involved in the formation of cell membrane components, ion (calcium) transporting activity, and the ergosterol biosynthesis KEGG pathway. Most of the genes whose expression decreased in response to zinc oxide were related to metabolic processes, catalytic activity, binding, redox processes, and oxidoreductase activity. Metabolic pathways, biosynthesis of secondary metabolites and biosynthesis of antibiotics were among the most important pathways that in the presence of zinc oxide nanoparticles had a decreased expression of the related genes. However, the down-regulated genes were often associated with metabolic processes, catalytic activity, binding, redox processes, oxidoreductase activity; and metabolic pathways, biosynthesis of secondary metabolites, and biosynthesis of antibiotics were the most vital pathways related to down-regulated DEGs. According to the evidence and obtained results, zinc oxide nanoparticles primarily affect the cell membrane and then induce oxidative stress through the production and accumulation of reactive oxygen species, and due to the inability of cells to detoxify ROSs mediated by down-regulation of the expression of involved genes in catalytic activity, the intracellular homeostasis is disrupted, which eventually leads to cell death. Overall, understanding the different layers of zinc oxide nanoparticles mechanism as a nanobiofungicide on *M. grisea* requires more effort based on the results of this study.

## Data Availability Statement

The datasets presented in this study can be found in online repositories. The names of the repository/repositories and accession number(s) can be found in the article/Supplementary Material.

## Author Contributions

AA contributed to the conceptualization and led the research. RG executed the experiments and original draft writing. MB and RG analyzed the RNA-seq data. MT and MB contributed to the new analytical tools and review and editing. All authors contributed to the article and approved the submitted version.

## Conflict of Interest

The authors declare that the research was conducted in the absence of any commercial or financial relationships that could be construed as a potential conflict of interest.

## Publisher’s Note

All claims expressed in this article are solely those of the authors and do not necessarily represent those of their affiliated organizations, or those of the publisher, the editors and the reviewers. Any product that may be evaluated in this article, or claim that may be made by its manufacturer, is not guaranteed or endorsed by the publisher.
