# Supplementary material for: RNA-Seq Analysis of Magnaporthe grisea Transcriptome Reveals the High Potential of ZnO Nanoparticles as a Nanofungicide
Source: Front Plant Sci. 2022 Jun 10;13:896283. doi: 10.3389/fpls.2022.896283 (PMC9230574; doi:10.3389/fpls.2022.896283)
Supplement: Supplementary file 2 [file Data_Sheet_1.pdf]

Supplementary Table 2

Table 1. The results of analysis of variance (ANOVA). Mean squares are presented for the effect of treatment (doses of ZnO nanoparticles) during 10 days of treatment.

S.O.V	df	Day1	Day2	Day3	Day4	Day5	Day6	Day7	Day8	Day9	Day10
Dose	6	16.50**	75.754**	178.281**	334.226**	537.809**	780.587**	1065.934**	1407.115**	1788.906**	2226.421**
error	14	0.012	0.012	0.607	0.702	0.702	0.405	0.667	0.676	1.071	1.083333

** indicating highly significant of the effect of treatments (dose of ZnO NPs) on growth of *M. grisae*. S.O.V: Source of variance.

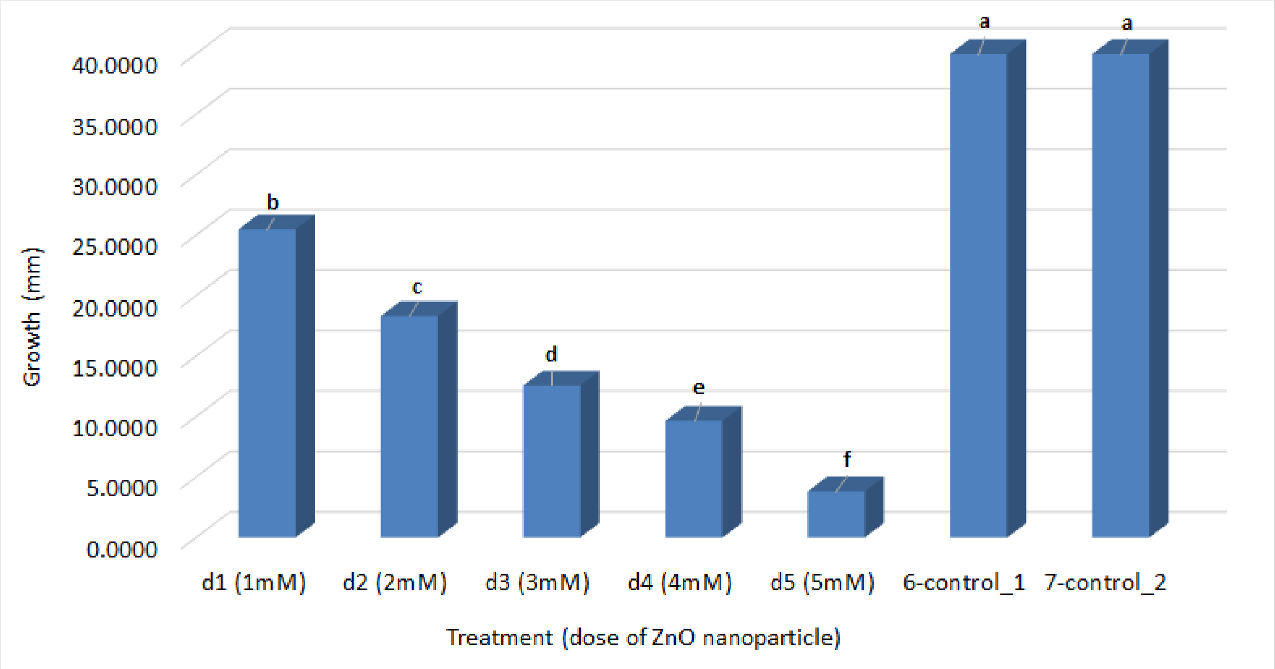

Figure 1. The results of Duncan's multiple range test for comparison of the effect of different doses of ZnO nanoparticles after 10th day. The significant differences between treatments are shown with different letters. As seen, dose d5 (5mM ZnO NPs) had the highest inhibitory effect on the growth of *Magnaporthe grisea*.
